# Medical Research

**Published:** 1920-04-17

**Authors:** 


### MEDICAL RESEARCH.

### A Great Step Forward.

It is announced in the Press that a new and important development has taken place with regard to medical research. A Charter has received the King's approval, and a warrant prepared accordingly, the significance of which will be to transform the existing Medical Research Committee into a corporate body styled the Medical Research Council, with an enlarged sphere of duty and with considerably enhanced responsibilities. The new Council will carry out its work under the aegis of a Committee of the Privy Council, whose constitution will be the Lord President of the Privy Council, the Minister for Health, the Secretary for Scotland, and the Chief Secretary for Ireland. The Medical Research Council thus becomes a permanent sub-committee of the Privy Council, and can enter into contracts, hold personal property and dispose of the same, including Parliamentary grants. The members of the Council will have direct access to the principal Ministers concerned in the constituent parts of the United Kingdom.

The advice of the Royal Society is to be taken in respect of the *personnel* of the Council, which at first is to consist of the existing Medical Research

**THE PUBLIC WELL-BEING**—*(continued)*.

Committee. Three members of the Council will retire at intervals of two years, and appointments to vacancies are to be made by the Supervising Committee of the Privy Council after consultation with the existing body itself, and with the President of the Royal Society. The *Morning Post* says the significance of the new development is apparent to all those who appreciate the essential importance of the endowment of scientific research. Henceforth the Medical Research Council as a permanent institution will be charged with the responsibility for the encouragement and maintenance of research, and it is obvious that, in order to fulfil the requirements of the Public Health, the Council will be enabled under its charter to provide for scientific research that support which has been so long lacking in this country. Hitherto scientific research has largely been carried on at private expense, and the whole position has been thoroughly unsatisfactory, notwithstanding the fact that upon the advance of science largely depends the present and future welfare of the country. This is a great step forward, and perhaps it may be taken as Mr. Balfour's executive reply to the deputation which called upon him some time ago appealing for a grant for the better remuneration of research workers. The new Council will presumably be able to deal with this point itself, for it will not be much use giving wide powers and big status without the means of utilising them for the benefit of the community.

